# Autofluorescence Imaging During Surgery for Orthopedic Infection: A Prospective Feasibility Case Series

**DOI:** 10.3390/jcm15208055

**Published:** 2026-10-16

**Authors:** Mari Babasiz, Jessica Köller, Tamaradoubra T. Tuburu, Andreas Christian Strauss, Elio Assaf, Max Jaenisch, Dieter C. Wirtz, Frank A. Schildberg

**Affiliations:** Department of Orthopedics and Trauma Surgery, University Hospital Bonn, University of Bonn, 53113 Bonn, Germany

**Keywords:** PJI, osteomyelitis, spondylodiscitis, VELscope, autofluorescence imaging

## Abstract

**Background/Objectives:** Intraoperative assessment of infected and nonviable tissue remains challenging in orthopedic surgery. This prospective case series evaluated the technical feasibility of VELscope^®^ autofluorescence imaging during surgery for orthopedic infection. **Methods:** Thirteen patients undergoing surgery for hip or knee periprosthetic joint infection or spondylodiscitis were included. Autofluorescence was assessed qualitatively before and after conventional debridement by the operating surgeon, without quantitative thresholds or independent, blinded image assessment. Microbiological and histopathological findings from selected sampled areas were summarized at the patient level. **Results:** Imaging was performed in all 13 cases without reported device-related complications. Altered autofluorescence was observed at baseline in all cases and was no longer observed after debridement judged macroscopically complete in 12 of 13 cases (92.3%; 95% CI 66.7–98.6). Sampling from areas with altered autofluorescence yielded culture growth in 11 of 13 cases (84.6%; 95% CI 57.8–95.7) and reported active inflammation in 10 of 13 cases (76.9%; 95% CI 49.7–91.8). **Conclusions:** Intraoperative autofluorescence imaging was technically feasible in this small series. The imaging and laboratory findings are exploratory. Diagnostic accuracy, reproducibility, debridement completeness, and clinical benefit remain to be established.

## 1. Introduction

Periprosthetic joint infection (PJI) is a serious complication of arthroplasty [1,2]. Despite advances in surgical techniques and wound management in endoprosthetic surgery, periprosthetic joint infection occurs in 0.5–2.3% of cases [1] and is associated with increased morbidity and mortality [3]. A comparable challenge arises in spondylodiscitis, where adequate debridement of infected and necrotic bone is essential for treatment success [4,5]. In septic orthopedic surgery, radical debridement with removal of all infected tissue, bone, and foreign material is the cornerstone of successful treatment [6,7]. Preoperative imaging using computed tomography or magnetic resonance imaging may assist in localizing infected structures [8], but no established routine method is available for the intraoperative visualization of infected or necrotic bone and soft tissue. Recent approaches such as dynamic contrast-enhanced fluorescence imaging for bone perfusion and bacteria-targeted fluorescent tracers have been explored to complement subjective intraoperative assessment [9,10], but these approaches are not established for routine clinical use.

The VELscope^®^ (Visually Enhanced Lesion Scope; LED Dental Inc., Vancouver, BC, Canada) is a portable device that visualizes tissue autofluorescence. Its use has been investigated in oral and maxillofacial surgery to differentiate vital from non-vital or necrotic tissue [11,12,13]. Fluorescence-guided surgery has been investigated most extensively in medication-related osteonecrosis of the jaw, where the intraoperative distinction between necrotic and viable bone is a well-recognized challenge. A review of 18 studies comprising 218 patients concluded that fluorescence-guided delineation of the resection margin is a promising method, while noting that available evidence remains limited, with only five prospective studies [11]. Tissue autofluorescence arises from endogenous tissue constituents and is distinct from fluorescence generated by administered labeling agents, such as tetracycline, or bacteria-targeted tracers.

Rupp et al. investigated VELscope^®^ imaging in ten patients with chronic fracture-related infection of the lower extremity and reported agreement between fluorescence findings and histological bone viability [7]. That study addressed viable versus necrotic bone rather than a site-specific diagnosis of infection. Viable bone may still contain inflammatory infiltrates. Therefore, preservation of fluorescence cannot be equated with absence of infection. The applicability of this approach to soft tissue and to distinguishing infected from uninfected sites remains uncertain.

The aim of this initial prospective feasibility study was to determine whether intraoperative VELscope^®^ autofluorescence imaging could be integrated into septic orthopedic revision surgery. Secondary exploratory objectives were to describe changes in visually assessed autofluorescence before and after conventional debridement and the microbiological and histopathological findings in sampled areas. The study was not designed to establish diagnostic accuracy, validate resection margins, or demonstrate improved clinical outcomes.

## 2. Materials and Methods

### 2.1. Study Design and Patient Population

**Figure 1:**
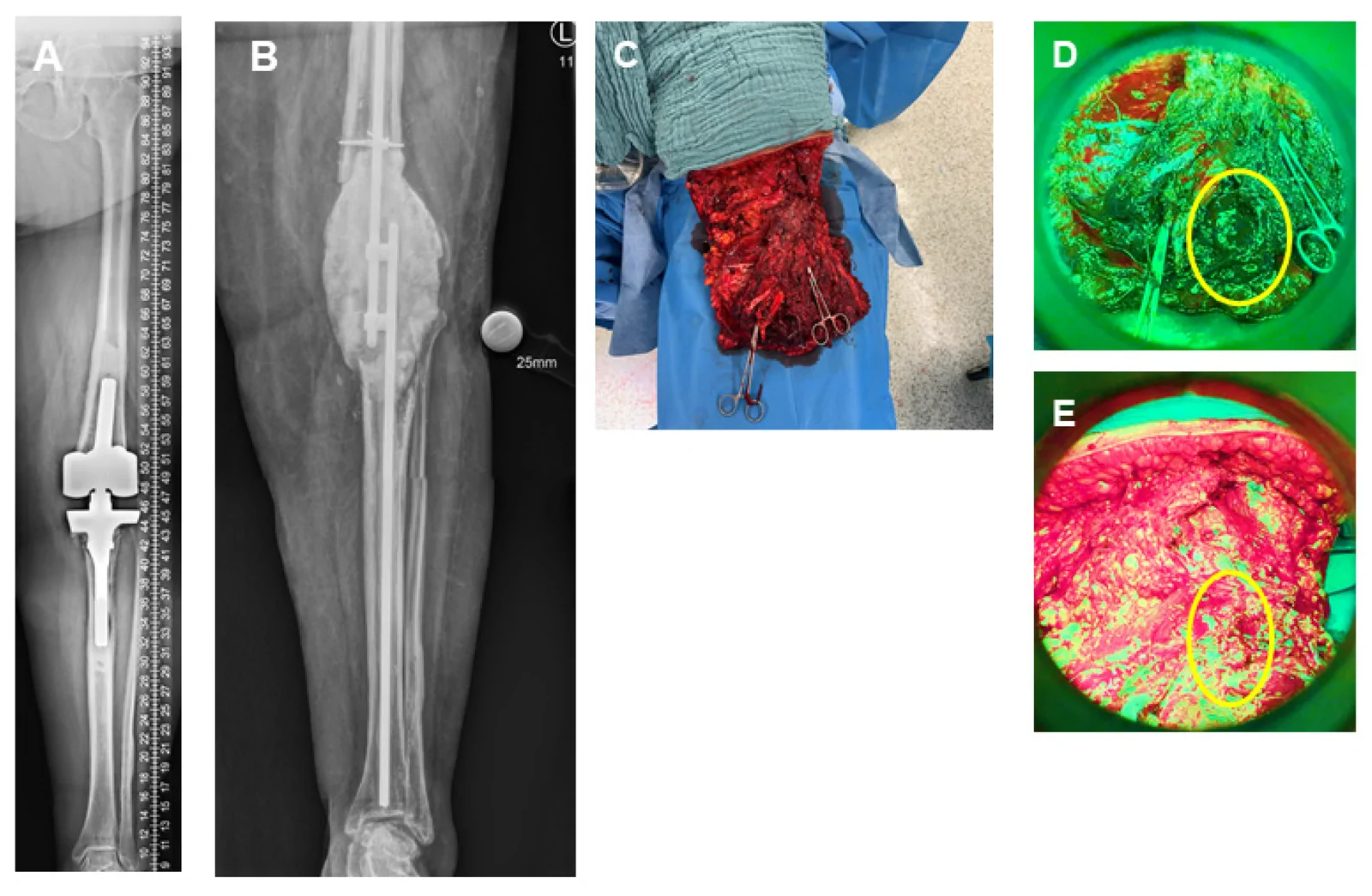
Female patient aged 58 years with persistent knee PJI despite revision procedures, prosthesis explantation, and static spacer implantation, leading to the decision for transfemoral amputation. The patient was included because of ongoing PJI during treatment with a spacer. All panels show the same patient. (**A**) Anteroposterior radiograph with the revision knee prosthesis in situ. (**B**) Anteroposterior radiograph after prosthesis explantation and static spacer implantation. (**C**) White-light view of the operative field before debridement. The visually suspected extent of infection was difficult to delineate. (**D**) Autofluorescence view before debridement, showing the area from which tissue was collected for laboratory analysis. The specimen consisted of soft tissue containing muscle and was obtained from the operative region shown in (**C**,**D**) before debridement. Imaging in panels (**C**–**E**) and the debridement were performed after completion of the transfemoral amputation. Circled regions indicate areas qualitatively considered altered. (**E**) Autofluorescence view after debridement judged macroscopically complete. The circled region was interpreted as showing no residual alteration. Staphylococcus epidermidis and active inflammation were reported in the sampled tissue. Color interpretation is descriptive, as stated in the Section 3, and does not establish infection or viability.

**Figure 2:**
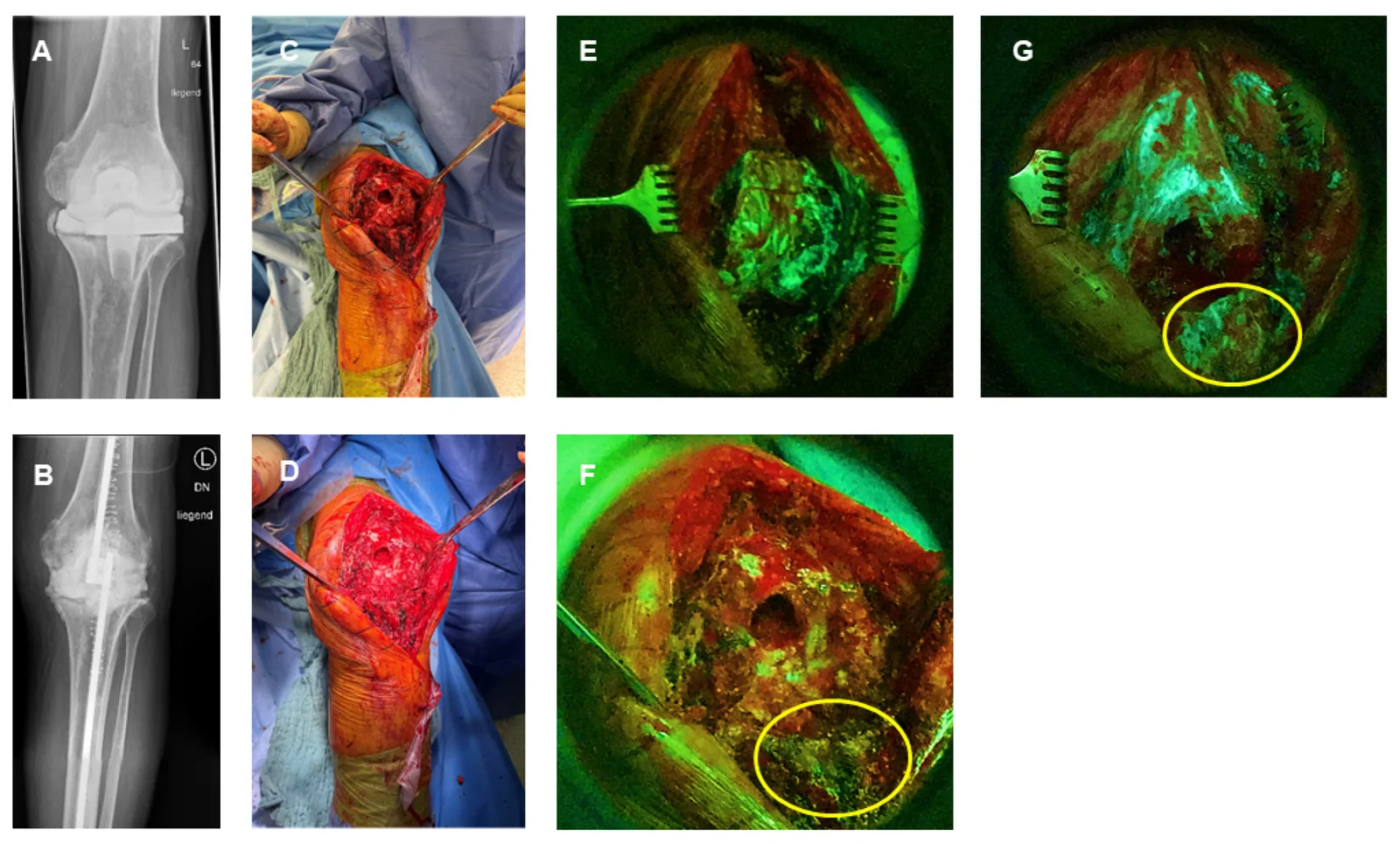
Patient aged 66 years with chronic knee PJI undergoing removal of a mobile spacer and implantation of a static spacer. All panels show the same patient. (**A**) Radiograph with the mobile spacer in situ. (**B**) Radiograph after mobile spacer removal and static spacer implantation. (**C**) Intraoperative white-light view before debridement. (**D**) Intraoperative white-light view after debridement. (**E**) Autofluorescence view before debridement with the mobile spacer still in situ. (**F**) Autofluorescence view after mobile spacer removal and before debridement; the circled area with qualitatively altered appearance was sampled for tissue analysis. (**G**) Autofluorescence view after debridement judged macroscopically complete; the circled region was interpreted as showing no residual alteration. Sampled tissue yielded Staphylococcus aureus; histopathology reported wear-particle-associated changes and fat necrosis without active inflammation. Color interpretation is descriptive as stated in the Section 3.

**Figure 3:**
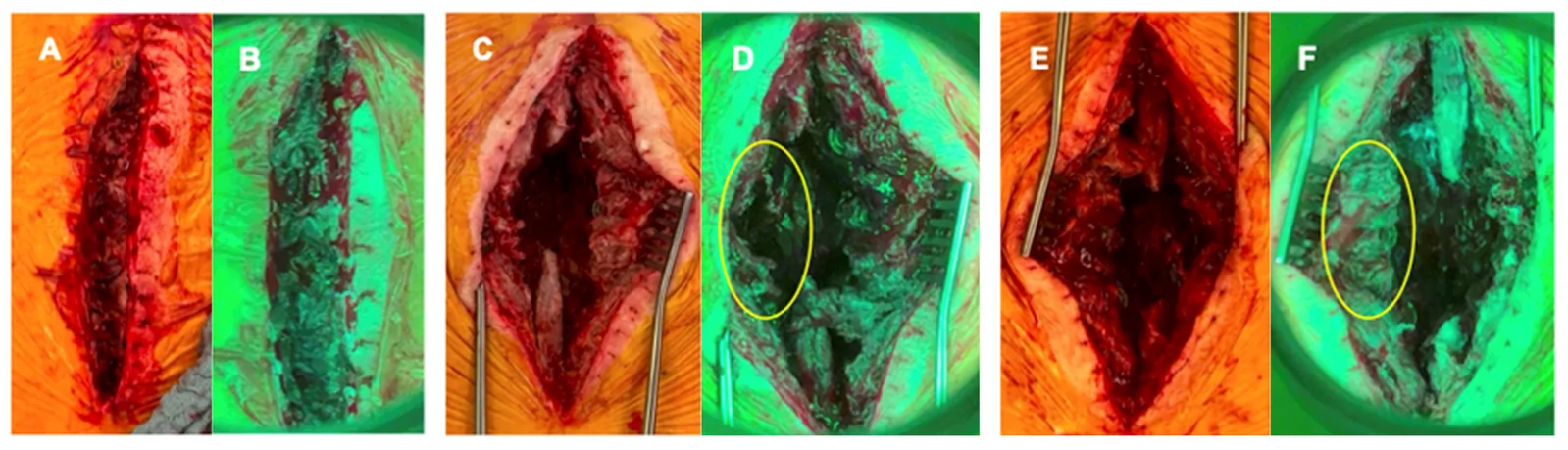
Patient P11, aged 64 years, undergoing surgery for native spondylodiscitis. All panels show the same patient. (**A**) White-light view at initial wound opening before debridement. (**B**) Corresponding baseline autofluorescence view. (**C**) White-light view after further exposure on the right side, before debridement. (**D**) Corresponding autofluorescence view; the circle identifies qualitatively altered appearance. (**E**) White-light view after debridement judged macroscopically complete. (**F**) Corresponding autofluorescence view with no residual alteration in the circled region. Case-level tissue findings for P11 included recovery of Candida albicans and Staphylococcus caprae and florid active inflammation. These case-level findings are not a spatially matched validation of each circled region. The patient with residual altered autofluorescence was P13 and is not illustrated in this figure. Color interpretation is descriptive as stated in the Section 3.

This prospective single-center feasibility case series was approved by the local institutional review board (reference 2024-20-BO; opinion dated 5 November 2025). Patients were prospectively enrolled between January and June 2026. Eligible patients were adults undergoing surgery for hip or knee periprosthetic joint infection (PJI), including procedures after resection arthroplasty or spacer implantation, or for native spondylodiscitis (Figure 1, Figure 2 and Figure 3). Informed consent was obtained. Enrollment was selective rather than consecutive; complete screening and exclusion counts were not available for analysis. All ten joint cases (P1–P10) met the “infection confirmed” category of the European Bone and Joint Infection Society (EBJIS) 2021 definition [14]. Spondylodiscitis was diagnosed from the clinical presentation, imaging, laboratory findings, and available microbiological results. All three spinal cases (P11–P13) involved native spondylodiscitis without pre-existing spinal instrumentation. Clinical infection diagnoses were distinct from assessment of individual sampled sites and were not site-specific reference standards for autofluorescence.

### 2.2. Surgical Procedure and Intraoperative Assessment

The operative sequence comprised exposure of the affected compartment and removal of implanted material where applicable, followed by baseline VELscope^®^ imaging (LED Dental Inc., Vancouver, BC, Canada) before debridement. Figure 2 also includes an initial view before spacer removal. Imaging was repeated after the operating surgeon judged debridement macroscopically complete under white-light illumination. During imaging, the operating room was darkened, and the device was directed at the exposed surgical field. According to the manufacturer, VELscope^®^ emits blue excitation light with a nominal spectral range of 400–460 nm [15]. Spectral output was not independently measured in this study. No tourniquet was used. Blood was removed by suction as thoroughly as possible, and images were obtained without active bleeding in the field. These measures did not constitute a quantitative correction for residual blood or optical attenuation. “Altered autofluorescence” denotes a qualitative visual assessment of an unusual fluorescence appearance, not a calibrated increase in fluorescence, bacterial fluorescence, or confirmed infection. No quantitative intensity measurements, predefined thresholds, standardized scoring system, or reproducible tissue-specific color criteria were used. Acquisition distance, exposure settings, and radiometric calibration were not available in the study dataset. Regions considered suspicious were marked at their borders using electrocautery before conventional white-light debridement. The surgeon therefore knew the baseline fluorescence findings and markings when deciding that debridement was complete. The assessments were neither independent nor blinded, and fluorescence may have influenced the extent of resection. Electrocautery-related thermal changes may also have affected subsequent optical appearance. Two surgeons performed the procedures: one performed all hip and knee procedures, and the other performed all spinal procedures. Autofluorescence was assessed by the operating surgeon; no independent image review was performed. Imaging did not establish the clinical diagnosis of infection. In each patient, three soft-tissue and three bone specimens were collected from areas classified as having altered autofluorescence. Two comparator specimens, one soft-tissue and one bone specimen, were obtained from areas classified as autofluorescence-negative. Thus, eight specimens were collected per patient, comprising six from altered areas and two comparators (104 specimens in total). Comparator sites were selected by the operating surgeon and were not independently established to be infection-free. Samples were submitted for microbiological culture and histopathology according to institutional procedures. Supplementary Table S1 reports culture growth/no growth for all 104 specimens by patient, region, tissue type, and sampled autofluorescence category. Labels are reporting labels rather than original laboratory identifiers. Exact anatomic subsites, collection time points, specimen-specific organisms, and specimen-specific histopathology were not available for analysis. Margins and transition zones were not systematically sampled. No additional sampling or debridement specifically targeting the residual altered area in P13 was performed.

Microbiological analysis used standard culture techniques with an incubation period of at least 14 days. For patient-level analysis of sampled altered areas, a case was classified as culture-positive if at least one specimen from those areas yielded microbial growth. Repeated recovery of the same organism from two or more specimens was not required. Possible contamination was not separately adjudicated, and all recovered organisms contributed to the descriptive case-level findings. Culture positivity therefore denotes organism recovery rather than proof of causative infection. Histopathology assessed inflammatory infiltrates and wear-particle-associated changes. Active inflammation was assessed by the reporting pathologist according to institutional routine histopathological criteria and extracted from the pathology reports as present or absent. A separate study-specific neutrophil-counting protocol was not used. The numerical neutrophil threshold, high-power-field count, and magnification used in routine reporting were not available in the study dataset. Reported Krenn/Morawietz categories [16] were recorded for periprosthetic tissue and were not used as a grading system for spinal tissue. Spinal specimens were assessed according to routine histopathological criteria for inflammation. Bone necrosis was not systematically quantified using a predefined threshold and was not a validated study endpoint. Fat necrosis does not establish bone necrosis. Pathologists and microbiologists were blinded to the autofluorescence findings.

VELscope^®^ autofluorescence findings were explored alongside microbiological and histopathological results from sampled sites. The results reported below summarize these findings at the patient level. They do not represent a systematic site-by-site diagnostic validation of the operative field.

### 2.3. Outcome Measures

The primary outcome was technical feasibility, defined descriptively by the ability to obtain intraoperative autofluorescence images at the planned stages of surgery. Reported device-related complications were recorded as an additional feasibility observation. Secondary exploratory outcomes were persistence of altered autofluorescence after debridement judged macroscopically complete and the proportions of patients with culture growth or reported active inflammation in sampled areas. These are patient-level descriptive findings, not diagnostic accuracy estimates or independent measures of debridement completeness. Imaging-related additional operating time was not quantified. Postoperative antimicrobial treatment and infection control at follow-up were not systematically analyzed as study outcomes.

### 2.4. Statistical Analysis

Given the exploratory feasibility design, no formal sample size calculation was performed, and analyses were descriptive. Age, BMI, operative time, estimated blood loss, and length of stay are summarized as median (range), calculated from the individual patient values overall and by anatomic subgroup. Categorical variables are presented as counts and proportions. Two-sided 95% confidence intervals for proportions were calculated using the Wilson score method. No hypothesis testing was performed. Analyses were carried out using GraphPad Prism, version 11 (GraphPad Software, Boston, MA, USA). The unit of analysis was the patient, with multiple selected samples contributing to each case-level result. Sensitivity, specificity, and predictive values were not estimated because sampling was selective and lacked a systematic, independently assessed classification of positive and negative sites. Preferential sampling of altered areas and a cohort selected for clinical infection limit diagnostic interpretation. Interobserver reproducibility and effects on clinical outcomes were not evaluated.

## 3. Results

Thirteen patients were included: five undergoing procedures for hip PJI, five for knee PJI, and three for spondylodiscitis. Case-level imaging and histopathological summaries and culture positivity for all 104 collected specimens were available. Median age was 71.9 years (range 57.1–87.7), and median body mass index was 32.7 kg/m^2^ (range 22.0–51.9). Ten of 13 patients (76.9%) were obese (BMI ≥ 30 kg/m^2^). Baseline and perioperative characteristics are summarized in Table 1.

Intraoperative VELscope^®^ imaging was performed in all 13 cases (100%) at the planned imaging stages, without reported device-related complications. The device could be integrated into the surgical workflow. Imaging-related additional operating time was not quantified, so no estimate of prolongation can be provided.

Baseline imaging before debridement showed areas of altered autofluorescence in all 13 cases. Representative operative and fluorescence views are presented in Figure 1, Figure 2 and Figure 3. Image colors are descriptive and are not diagnostic categories: green denotes visible fluorescence, dark areas denote reduced or absent visible fluorescence, and red, orange, or magenta denote the recorded color appearance. None of these appearances independently establishes infection or tissue viability, and differences between panels should not be interpreted quantitatively.

After debridement judged macroscopically complete, the previously observed autofluorescence alteration was no longer observed in 12 of 13 cases (92.3%; 95% CI 66.7–98.6). This denotes disappearance of the recorded alteration, not loss of all tissue fluorescence. A residual alteration persisted in P13 (1/13; 7.7%; 95% CI 1.4–33.3), a patient undergoing surgery for spondylodiscitis. Image acquisition in the affected spinal region was technically difficult, limiting visual assessment of the residual alteration. No additional debridement of the residual area was performed because the spinal anatomy limited the extent of further tissue removal. VELscope imaging was used as an adjunct to evaluate technical feasibility. The residual optical finding was not used as a diagnostic indication for additional resection. No additional targeted specimen was collected from the residual area. Laboratory and aggregate imaging findings are summarized in Table 2 and Table 3.

The specimen-level culture results are presented in Supplementary Table S1, with all eight specimens listed separately for each of the 13 patients. Of the 78 specimens from altered areas, 59 yielded growth and 19 showed no growth. These specimen counts are descriptive and are clustered within patients. Of the 26 comparator specimens, 25 showed no growth and one soft-tissue comparator specimen from P13 yielded growth. All 13 bone comparator specimens showed no growth. Soft-tissue comparator specimens showed no growth in P1–P12. A comparator denotes the autofluorescence category of the sampled site, not an independently established infection-free control.

Four cases illustrate differing relationships between altered autofluorescence, culture growth, and reported active inflammation. In P5 (hip), culture showed no growth while histopathology reported chronic active inflammation. In P9 (knee), altered autofluorescence was accompanied by no culture growth and no reported active inflammation. P9 had confirmed PJI according to the EBJIS 2021 criteria and received vancomycin before specimen collection. These negative sample findings do not establish the absence of clinical infection or a false-positive imaging result. Antibiotic pretreatment may have influenced culture yield, but its effect in this case cannot be determined. In P7 (knee) and P13 (spine), Staphylococcus aureus was recovered without reported active inflammation. Wear-particle-associated changes and fat necrosis were present in P7. Culture growth and histopathology assess different properties and should not be treated as interchangeable reference standards.

## 4. Discussion

This prospective single-center case series demonstrates the technical feasibility of integrating VELscope^®^ autofluorescence imaging into surgery for orthopedic infection. No device-related complications were reported in the 13 cases, although this small sample does not establish safety. Altered autofluorescence was observed at baseline in all cases and was no longer observed after debridement judged macroscopically complete in 12 of 13 cases. These observations describe qualitative changes in optical appearance. They do not provide independent confirmation of tissue viability, infection clearance, or debridement completeness. Whether the technique improves surgical decision-making or clinical outcomes remains unknown.

Residual altered autofluorescence occurred in P13, a patient undergoing surgery for spondylodiscitis. P13 had case-level recovery of Staphylococcus aureus and no reported active inflammation. Florid active inflammation was reported in P11, whose altered autofluorescence resolved after debridement and who is shown in Figure 3. In P13, image acquisition was technically difficult and spinal anatomy limited further tissue removal. No additional debridement or targeted specimen collection was performed in the residual area. Its optical appearance therefore cannot establish residual infection or be spatially validated against the case-level laboratory findings.

Biologically, altered tissue autofluorescence may reflect changes in endogenous fluorophores and the optical properties of the surrounding tissue. Structural components such as collagen and elastin, as well as metabolic cofactors including NAD(P)H and flavins, contribute to intrinsic tissue fluorescence [17,18,19]. Changes in cellular metabolism, extracellular matrix composition, and tissue integrity may therefore modify the observed fluorescence signal. Pathological processes such as inflammation, tissue injury, and necrosis may be associated with these alterations, while optical absorption and scattering, particularly in the presence of blood, can further influence the visual appearance [17,18]. Importantly, these mechanisms were not directly investigated in the present study. Consequently, qualitative autofluorescence changes cannot be attributed to a specific biological process or interpreted as a reliable marker of infection.

The VELscope^®^ may warrant further investigation as an adjunct in the surgical management of orthopedic infection. Although complete debridement and removal of infected and necrotic tissue are central determinants of treatment success, the extent of resection currently relies largely on the surgeon’s judgment, and no established routine method is available for intraoperative delineation of infected or nonviable bone and soft tissue [1]. VELscope^®^ has been investigated in oral and maxillofacial surgery for the delineation of osteonecrosis [11,13]. In an initial clinical series of chronic fracture-related infection, Rupp et al. reported agreement between VELscope^®^ fluorescence findings and histological assessment of viable versus necrotic bone [7]. This finding does not establish the ability to distinguish infected from uninfected tissue. In periprosthetic joint infection, intraoperative approaches such as fluorescent tetracycline bone labeling and bacteria-targeted fluorescent tracers have been explored to identify nonviable or infected bone and to guide debridement, but these remain preliminary and are not established for routine clinical use [19,20,21]. Comparison with bleeding bone (the “paprika sign”) was not systematically evaluated. Using altered autofluorescence alone to define a resection margin could lead to unnecessary removal of viable tissue, particularly in revision arthroplasty. The present findings do not justify fluorescence-defined resection margins.

The recovered organisms included *coagulase-negative staphylococci*, *Corynebacterium species*, *Micrococcus luteus*, *and Candida species*. Organism recovery requires interpretation alongside specimen number, repeated recovery, antibiotic exposure, histopathology, and the clinical course. Possible contamination was not separately adjudicated in this study. The culture-positive numerator included cases with any growth from sampled altered areas, without requiring repeated recovery of an organism. Consequently, potentially contaminating isolates may have contributed to the descriptive yield. P9 had confirmed PJI according to the EBJIS 2021 criteria and received vancomycin before specimen collection, despite no culture growth or reported active inflammation in the selected samples. This discordance does not establish a false-positive imaging result. Antibiotic pretreatment may have influenced culture yield. Altered autofluorescence alone remains insufficient to establish infection at a sampled site. The laboratory results provide exploratory context but do not validate specificity or infection boundaries.

This study has important limitations. It is a small, heterogeneous, single-center feasibility series with selective rather than consecutive enrollment, creating a risk of selection bias. Complete screening and exclusion counts were unavailable. Although all joint cases were clinically classified as confirmed PJI, the supporting EBJIS criteria were not tabulated individually. The three spinal cases were diagnosed from clinical, imaging, laboratory, and microbiological findings rather than a separately prespecified research case definition. Sampling favored altered areas, with six specimens from altered areas and two surgeon-selected comparators per patient. Margins and transition zones were not systematically sampled, and exact spatial and temporal linkage, specimen-specific organisms, and specimen-specific histopathology were unavailable for analysis. These limitations preclude valid diagnostic-accuracy estimates and verification of resection margins. Fluorescence assessment was qualitative, without calibrated intensity measurements or tissue-specific thresholds. Blood was removed by suction, and no tourniquet was used, but residual blood, hematoma, tissue composition, fat necrosis, cement or other debris, thermal changes, and acquisition conditions may affect optical appearance; their individual effects were not assessed. The surgeon knew the baseline findings and electrocautery markings before judging debridement complete, so both information and thermal effects may have influenced the later assessment. Despite blinded laboratory assessment, independent or blinded image review was not performed, and interobserver reproducibility remains unknown. Possible culture contamination was not separately adjudicated, and a single positive specimen sufficed for case-level culture positivity. Inflammation was assessed according to institutional routine histopathological criteria, but numerical neutrophil thresholds, field counts, and magnification were not available in the study dataset. Bone necrosis was not a validated endpoint. The residual alteration in P13 was not further debrided because of anatomic constraints and was not sampled separately. Its microbiological and histopathological status therefore remained unverified. Technically difficult acquisition in this spinal region limited interpretation; restricted access and exposure may also limit use in other spinal procedures. Normal-appearing fluorescence does not establish absence of infection. Absence of reported device-related complications in 13 patients does not establish safety. Postoperative treatment and follow-up were not systematically analyzed, and effects on infection eradication, morbidity, mortality, or other clinical outcomes remain unknown.

A subsequent prospective diagnostic-validation study should incorporate standardized digital image acquisition, quantitative or semi-quantitative fluorescence analysis, predefined regions of interest, and systematic spatially matched sampling from the centers and margins of areas with altered autofluorescence, their transition zones, and predefined autofluorescence-negative sites. Independent microbiological and histopathological assessment, blinded image review by observers not involved in surgery, and evaluation of interobserver agreement would be required. Prespecified thresholds should be validated independently, and analyses should account for multiple samples within each patient. A separate comparative evaluation would be needed to determine whether the technique improves surgical decision-making or clinical outcomes.

## 5. Conclusions

Intraoperative VELscope^®^ autofluorescence imaging was technically feasible in this small series of septic orthopedic revision procedures. Altered autofluorescence resolved after conventional debridement in 12 of 13 cases, but this observation does not demonstrate complete removal of infected or necrotic tissue. The microbiological and histopathological findings are exploratory and hypothesis-generating. Diagnostic accuracy, reproducibility, spatial delineation of resection margins, and clinical benefit require subsequent prospective validation.

## Figures and Tables

**Table 1:** Baseline and perioperative characteristics of the study cohort.

Characteristic	Overall (*n* = 13)	Hip (*n* = 5)	Knee (*n* = 5)	Spine (*n* = 3)
Age, years, median (range)	71.9 (57.1–87.7)	71.9 (57.1–87.7)	69.5 (58.0–87.1)	74.3 (64.0–80.0)
Sex m/f	6/7	1/4	2/3	3/0
Body-mass index, kg/m^2^, median (range)	32.7 (22.0–51.9)	32.7 (32.3–39.8)	32.7 (25.1–51.9)	30.4 (22.0–33.7)
Obesity (BMI ≥ 30)-*n*/N (%)	10/13 (76.9)	5/5 (100)	3/5 (60.0)	2/3 (66.7)
Operative time, min, median (range)	136 (40–238)	142 (47–206)	162 (123–238)	61 (40–115)
Estimated blood loss, mL, median (range)	300 (100–700)	300 (200–650)	500 (200–700)	200 (100–300)
Length of stay, days, median (range)	14.0 (7–20)	13.0 (7–20)	14.0 (12–17)	13.0 (11–20)

Age, BMI, estimated blood loss, operative time, and length of stay are presented as median (range), calculated from individual patient values. Categorical values are *n*/N (%).

**Table 2:** Microbiological and histopathological findings per case.

Case	Region	Microbiological Result	Recovered Species Pattern	Histopathological Pattern	Comparator Culture Soft Tissue/Bone	Clinical Diagnosis
**P1**	Hip	*Citrobacter koseri*, *Micrococcus luteus*, *Candida parapsilosis*	Multiple species	Chronic active inflammation	No growth/No growth	Confirmed PJI (EBJIS 2021)
**P2**	Hip	*Enterococcus faecium*	One species	Chronic active inflammation; mixed type (Morawietz III)	No growth/No growth	Confirmed PJI (EBJIS 2021)
**P3**	Hip	*Staphylococcus epidermidis*	One species	Chronic active inflammation with wear particles (Morawietz III)	No growth/No growth	Confirmed PJI (EBJIS 2021)
**P4**	Hip	*Escherichia coli*, *Enterobacter cloacae*	Multiple species	Chronic active inflammation with wear particles (Morawietz III)	No growth/No growth	Confirmed PJI (EBJIS 2021)
**P5**	Hip	No growth	No growth	Chronic active inflammation	No growth/No growth	Confirmed PJI (EBJIS 2021)
**P6**	Knee	*Corynebacterium striatum*	One species	Chronic active inflammation with wear particles (Morawietz III)	No growth/No growth	Confirmed PJI (EBJIS 2021)
**P7**	Knee	*Staphylococcus aureus*	One species	Wear particle (Morawietz I), fat necrosis, no active inflammation	No growth/No growth	Confirmed PJI (EBJIS 2021)
**P8**	Knee	*Staphylococcus epidermidis*	One species	Florid active inflammation	No growth/No growth	Confirmed PJI (EBJIS 2021)
**P9**	Knee	No growth	No growth	No active inflammation	No growth/No growth	Confirmed PJI (EBJIS 2021)
**P10**	Knee	*Streptococcus agalactiae*, *Staphylococcus aureus*	Multiple species	Chronic active inflammation	No growth/No growth	Confirmed PJI (EBJIS 2021)
**P11**	Spine	*Candida albicans*, *Staphylococcus caprae*	Multiple species	Florid active inflammation	No growth/No growth	Native spondylodiscitis; no prior instrumentation
**P12**	Spine	*Staphylococcus hominis*, *S. epidermidis*, *S. caprae*, *Corynebacterium aurimucosum*	Multiple species	Chronic active inflammation	No growth/No growth	Native spondylodiscitis; no prior instrumentation
**P13**	Spine	*Staphylococcus aureus*	One species	No active inflammation	Positive/No growth	Native spondylodiscitis; no prior instrumentation

Clinical diagnoses are reported separately from sampled-site laboratory findings. All joint cases (P1–P10) had confirmed PJI according to EBJIS 2021; P11–P13 had native spondylodiscitis without pre-existing spinal instrumentation. Microbiological entries describe organisms recovered per case, without separate adjudication of contamination or causative infection. Culture positivity required growth in at least one specimen from an altered area; repeated recovery was not required. The recovered-species pattern describes species count, not proof of polymicrobial causative infection. Histopathological findings and Krenn/Morawietz categories are reproduced from the pathology reports. Inflammation was assessed using institutional routine histopathological criteria; study-level neutrophil counts were not available. All patients had baseline altered autofluorescence. After debridement, altered autofluorescence was no longer observed in P1–P12 and persisted in P13. Comparator culture results are reported separately for soft tissue and bone; all specimen-level culture results are listed in Supplementary Table S1.

**Table 3:** Descriptive autofluorescence findings and microbiological and histopathological results at the patient level.

Parameter	*n*/N (%)
Cases with baseline altered autofluorescence	13/13 (100)
No residual altered autofluorescence after debridement judged macroscopically complete	12/13 (92.3; 95% CI 66.7–98.6)
Residual altered autofluorescence after debridement judged macroscopically complete	1/13 (7.7; 95% CI 1.4–33.3)
Culture growth in sampled altered areas	11/13 (84.6; 95% CI 57.8–95.7)
Reported active inflammation in sampled altered areas	10/13 (76.9; 95% CI 49.7–91.8)
Culture growth and reported active inflammation	9/13 (69.2%; 95% CI 42.4–87.3)
More than one microbial species recovered per case	5/13 (38.5%; 95% CI 17.7–64.5)
Fungal species recovered	2/13 (15.4%; 95% CI 4.3–42.2)

All proportions are descriptive patient-level findings from selectively sampled areas. Culture growth and active inflammation are not diagnostic confirmation rates or estimates of positive predictive value. Wilson 95% confidence intervals are provided where shown. Residual altered autofluorescence occurred in P13. More than one recovered species does not by itself establish polymicrobial causative infection. P11 shown in Figure 3 had no residual alteration.

## Data Availability

The original contributions presented in this study are included in the article. Further inquiries can be directed to the corresponding author.

## Supplementary Materials

The following supporting information can be downloaded at: https://www.mdpi.com/article/10.3390/jcm15208055/s1, Supplementary Table S1: Specimen-level microbiological culture results for 104 samples collected from 13 patients undergoing septic orthopedic revision surgery, stratified by patient, anatomical region, tissue type, and intraoperative autofluorescence category.
