# Supplementary material for: Autofluorescence Imaging During Surgery for Orthopedic Infection: A Prospective Feasibility Case Series
Source: J Clin Med. 2026 Oct 16;15(20):8055. doi: 10.3390/jcm15208055 (PMC13705935; doi:10.3390/jcm15208055)
Supplement: Supplementary file 1 [file jcm-15-08055-s001.zip › jcm-4556330-supplementary.pdf]

Journal of
Clinical Medicine

MDPI

Supplementary Table S1 Culture results for all 104 collected specimens

Case	Re-gion	T1	T2	T3	B1	B2	B3	CT	CB
**P1**	Hip	+	+	−	+	+	+	−	−
**P2**	Hip	+	+	+	+	−	+	−	−
**P3**	Hip	+	+	+	+	−	+	−	−
**P4**	Hip	+	+	+	+	+	+	−	−
**P5**	Hip	−	−	−	−	−	−	−	−
**P6**	Knee	+	+	+	+	+	+	−	−
**P7**	Knee	+	−	+	+	+	+	−	−
**P8**	Knee	+	+	−	+	+	+	−	−
**P9**	Knee	−	−	−	−	−	−	−	−
**P10**	Knee	+	+	+	+	+	+	−	−
**P11**	Spine	+	+	+	+	+	−	−	−
**P12**	Spine	+	+	+	+	+	+	−	−
**P13**	Spine	+	+	+	+	+	−	+	−

T1–T3: soft-tissue specimens; B1–B3: bone specimens from altered areas. CT/CB: soft-tissue/bone comparator from autofluorescence-negative areas. +: growth; −: no growth. Each cell denotes one specimen. Labels are assigned for reporting. Specimen-level linkage limitations are detailed in Sections 2.2 and 4.
